# The U-Shaped Association between Sleep Duration, All-Cause Mortality and Cardiovascular Risk in a Hispanic/Latino Clinically Based Cohort

**DOI:** 10.3390/jcm12154961

**Published:** 2023-07-28

**Authors:** Mario Henríquez-Beltrán, Jorge Dreyse, Jorge Jorquera, Jorge Jorquera-Diaz, Constanza Salas, Isabel Fernandez-Bussy, Gonzalo Labarca

**Affiliations:** 1 Escuela de Kinesiología, Facultad de Salud, Universidad Santo Tomás, Los Angeles 4440000, Chile; eliashbm@hotmail.com; 2 Centro de Enfermedades Respiratorias, Clínica Las Condes, Facultad de Medicina Universidad Finis Terrae, Santiago 7591047, Chile; jidreyse@gmail.com (J.D.); jjorquera@hotmail.com (J.J.); csalasc@clinicalascondes.cl (C.S.); 3 Universidad Favarolo, Buenos Aires C1079ABE, Argentina; jorgejorquera19@gmail.com; 4 Universidad Catolica Argentina, Buenos Aires C1107AFB, Argentina; izzyfb@gmail.com; 5 Department of Clinical Biochemistry and Immunology, Faculty of Pharmacy, University of Concepción, Concepción 4070112, Chile; 6 Division of Sleep Medicine, Beth Israel Deaconess Medical Center, Harvard Medical School, 330 Brookline Ave., Boston, MA 02215, USA

**Keywords:** sleep, short sleep, prolonged sleep, OSA, cardiovascular risk, mortality, Hispanic, Latino

## Abstract

Sleep is essential for life, and inappropriate sleep duration patterns may lead to chronic consequences regarding human health. Several studies have confirmed the presence of a U-shaped association between sleep duration and mortality. Moreover, many consequences related to cardiometabolic aspects have been suggested in patients with abnormal sleep durations. In this study, we analyzed the associations between sleep duration, total sleep time (TST), the risk of all-cause mortality, and 10-year cardiovascular risk in a cohort of patients at a sleep medicine center in Santiago, Chile. We conducted a prospective cohort study of patients (SantOSA). A short TST was defined as ≤6 h, a normal TST as 6 to 9 h, and a long TST as ≥9 h. Adjusted hazard ratios (aHRs) for all-cause mortality were calculated. A cross-sectional analysis between TST and 10-year cardiovascular risk (calculated using the Framingham 2008 formula) was determined using logistic regression models. A total of 1385 subjects were included in the results (78% male; median age: 53, interquartile range (IQR): 42–64 years; median BMI: 29.5, IQR: 16.7–33.1). A total of 333 subjects (24%) reported short TSTs, 938 (67.7%) reported normal TSTs, and 114 (8.3%) reported long TSTs. In the fully adjusted model, the association remained significant for short (aHR: 2.51 (1.48–4.25); *p*-value = 0.01) and long TSTs (aHR: 3.97 (1.53–10.29); *p*-value = 0.04). Finally, a U-shaped association was found between short and long TSTs, with an increase in cardiovascular risk at 10 years. Compared with normal TSTs, short (≤6 h) and long (≥9 h) TSTs were significantly associated with all-cause mortality and increased 10-year cardiovascular risk.

## 1. Introduction

Sleep is essential for life, and has been linked to mortality and an ever-growing number of diseases [1,2,3]. The American Heart Association (AHA) has recognized the importance of sleep and its association with cardiovascular disease (CVD) risk [4,5]. Healthy behaviors have beneficial impacts in reducing cardiovascular disease and mortality [6,7]. Several studies have suggested an association between cardiovascular disease and unhealthy lifestyles, such as sedentary behavior [8], unhealthy dietary behavior [9], and unhealthy sleep patterns [10].

According to the American Academy of Sleep Medicine (AASM)’s consensus, 7–8 h of sleep per night are appropriate for optimal health in the adult population [11]. Short and long sleepers are characterized by sleep pattern durations of less than 7 h and ≥9–10 h, respectively [12]. Both sleep pattern durations have been associated with adverse health outcomes, such as cardiometabolic disease, depression, and increased risk of death [13,14]. Similar occurrences have been observed with obstructive sleep apnea (OSA) in the adult population, in which obstructive episodes, hypoxemic episodes, and sympathetic activity lead to hypertension and cardiovascular compromise, increasing the risks of cardiac events and mortality [3,15,16,17,18]. The risk of presenting adverse health events is associated with daily sleep pattern duration, and unhealthy sleep is associated with increased cardiovascular risk, which has been reaffirmed by the AHA [19]. The association between cardiovascular risk and sleep health has been described in the Latin American population; however, the population studied has mainly been the Hispanic/Latino community that belongs to North America [20,21,22,23]. Additionally, the evidence concerning sleep pattern durations in the Hispanic/Latino population located in their country of origin is scarce. Our primary objective was to determine the association between sleep duration and the incidence of all-cause mortality in a cohort of patients at a sleep medicine center in Santiago, Chile. As a secondary objective, we determined the relationship between sleep duration and cardiovascular risk.

## 2. Materials and Methods

### 2.1. Study Design

We performed a secondary analysis of a prospective clinically-based cohort study (SantOSA), registry number ISRCTN62293645, of the consequences of OSA among patients, having been derived for clinical evaluation of OSA in a tertiary center in the urban area of Santiago, Chile. This cohort was established in 2009 and included adults aged ≥18 years [24,25,26]. Moreover, all missing data were excluded.

To date, there are records of 1928 participants who consulted with the sleep center in the last 12 years. Each participant signed an informed consent before inclusion. This study was conducted in accordance with the Declaration of Helsinki and approved by the Institutional Review Board of Clinica Las Condes (protocol number 10; 9 March 2018).

#### Sleep Assessment

At baseline, a standardized symptom questionnaire was applied to evaluate each participant’s sleep schedule, degree of daytime sleepiness, snoring intensity, witnessed apnea, insomnia, episodes of nocturnal suffocation, and morning headache. In addition, we collected data from the Spanish version of the Epworth Sleepiness Scale (ESS) [19,20].

To conduct a home sleep apnea test (HSAT), we used a validated type 3 sleep test (Embletta^®^ MPR device, Natus Medical Incorporated, Middleton, WI, USA) that included nasal pressure assessment (measurement of airflow), thoracic and abdominal inductance plethysmography, body position assessment, audio assessment via microphone, and pulse oximetry. Compared to polysomnography, 92.4% sensitivity and 85.7% specificity in diagnosing OSA have been reported for this device [21]. The HSAT analysis was performed manually by a respiratory disease specialist (author: J.J.) blinded to the clinical history of the subjects. Respiratory events were scored according to the current AASM guidelines; apnea was defined as airflow absence for 10 s, while hypopnea was defined as an airflow reduction of 30% associated with a drop in Spo2 by 3%. We also extracted data regarding the respiratory disturbance index (RDI), and times under 90% (T90%) were recorded. Finally, OSA diagnoses were achieved according to the RDI. An RDI of <5 events/h signified non-OSA; between 5 and 14 events/h signified moderate OSA, between 15 and 30 events/h signified moderate OSA, and ≥30 events/h signified severe OSA.

### 2.2. Primary Exposure: Total Sleep Time

We defined self-reported total sleep time, obtained through a questionnaire administered during each clinic visit, as the corresponding answer to the question “During the past month, how many hours of actual sleep time did you get at night” (different from the number of hours spent in bed). For this study’s purposes, sleep times were categorized as short sleep patterns (defined as TSTs of ≤6 h), normal sleep (defined as TSTs between 6 and 9 h), and long sleep (defined as TSTs of ≥9 h).

### 2.3. Covariates

Data from clinical and demographic variables were extracted from medical records, self-reported anthropometric variables (height (cm) and weight (Kg)) were measured at baseline, and the body mass index (BMI; Kg/m^2^) was calculated for each subject. In addition, the ESS and the home sleep test, using an Embletta, were used for all participants. The sleep study was manually scored according to the AASM guidelines, and data regarding the RDI and times under 90% (T90%) were recorded. Finally, OSA diagnoses were identified according to the RDI. An RDI of <5 events/h signified non-OSA, between 5 and 14 events/h signified moderate OSA, between 15 and 30 events/h signified moderate OSA, and ≥30 events/h signified severe OSA.

### 2.4. Outcome Assessment

As a primary outcome, we identified the mortality risks from all causes by inspecting the death certificates available in the Chilean civil registry (www.registrocivil.cl, last access 26 April 2022). As a secondary outcome, we analyzed the association between duration of sleep and cardiovascular risk at 10 years using the Framingham 2008 formula, which was calculated using the R software “CVRisk” package (R-project; https://www.r-project.org/, version 2022.12.0+353). We chose this method because it did not require a laboratory for estimation [27].

### 2.5. Statistical Analysis

Associations between the different interest categories were presented as medians and IQRs for continuous variables and frequencies (as % values) for categorical variables. Additionally, intragroup differences were analyzed using chi-square tests for categorical variables and ANOVAs for continuous variables. To determine the associations between the different categories of total sleep time and the risk of all-cause mortality, we performed three incremental Cox regression models: Model 1 (unadjusted), Model 2 (adjusted for the baseline cardiovascular risk), and Model 3 (Model 2 adjusted for the rate of respiratory events). A proportional hazards assumption (a test to measure whether an effect is not proportional to time) was carried out using the Schoenfeld residuals scaled against the transformed time. Finally, two complementary analyses were developed to establish the association between sleep patterns/TST and cardiovascular risk. First, a multivariable linear regression was performed; then, to establish the U-shaped relationship, a multivariate linear regression model of three was performed. Polynomial degrees established the coefficient that corresponded to each hour and its respective association with cardiovascular risk. All analyses were performed using R software (R-project), and statistically significant values were established as *p* < 0.05.

## 3. Results

**Figure 1:**
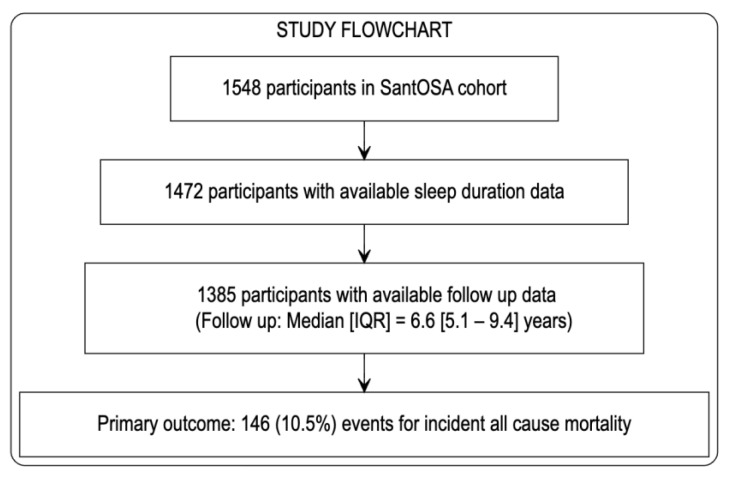
Study flowchart.

The study flowchart is available in Figure 1. From 1548 subjects, we excluded 163 (10.5%) due to missing values, repeated sleep tests, and unavailable follow-up data. Finally, a total of 1385 subjects were included in further analysis. Of these subjects, 333 (24%) reported short sleep times, 938 (67.7%) reported normal sleep times, and 114 (8.3%) reported long sleep times. The median age of the sample was 53 (IQR: 42–64) years, the median BMI was 29.5 (IQR: 16.7–33.1); the median ESS value was 8.0 points (IQR: 5–12), the median TST was 6.5 h (IQR: 6–7.5), the median AHI value was 18.7 events/h (IQR: 8.7–35.6), the median T90% value was 3.6 (IQR: 0.4–17.6), and the median/IQR Framingham CVD risk was 13.6% (IQR: 6.7–27.2). Additionally, the causes of mortality presented the following prevalences: 26.4% for CVD, 19.4% for cancer, and 54% for other causes. The baseline characteristics of the cohort, stratified by TST, are shown in Table 1.

### 3.1. Association between Total Sleep Time and All-Cause Mortality

**Figure 2:**
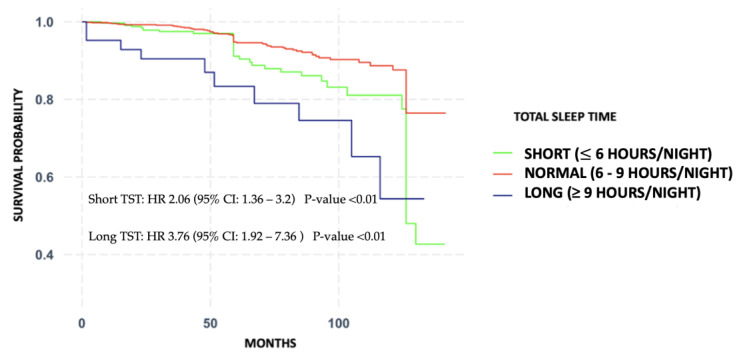
Kaplan—Meier curves showing the probability of survival in three categories: short sleep patterns (≤6 h/night), normal sleep patterns (6–9 h/night), and prolonged sleep patterns (≥9 h/night).

During the median follow-up of the cohort at 6.6 years (IQR: 5.1–9.4), we found 146 deaths (10.5%). Summaries of the non-adjusted and adjusted models of sleep patterns (TSTs) and mortality risk are shown in Table 2. The fully adjusted model showed an increased association between short sleep patterns and all-cause mortality risk (HR = 2.51; 95% CI: 1.48–4.25; *p*-value = 0.01) when compared with normal sleep patterns, and prolonged sleep patterns were also associated with all-cause mortality (HR = 3.97; 95% CI: 1.53–10.29; *p*-value = 0.04). The Schoenfeld residuals scaled against the transformed times showed no significant effect (*p*-value = 0.88). The unadjusted Kaplan–Maier survival curves, arranged by TST category, are shown in Figure 2.

### 3.2. Association between Total Sleep Time and CVD Risk

**Figure 3:**
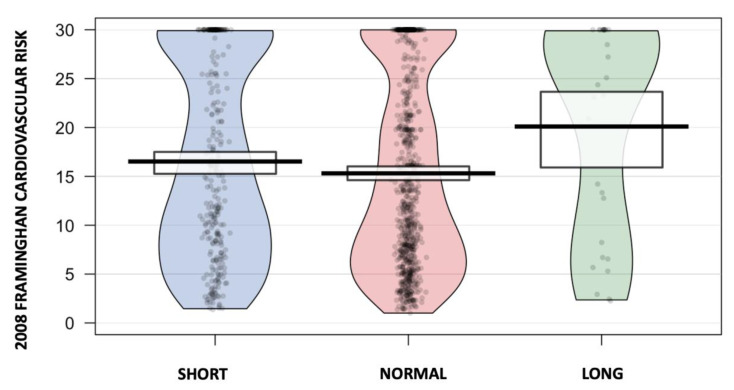
Individual distribution of the different groups evaluated according to TST category.

**Figure 4:**
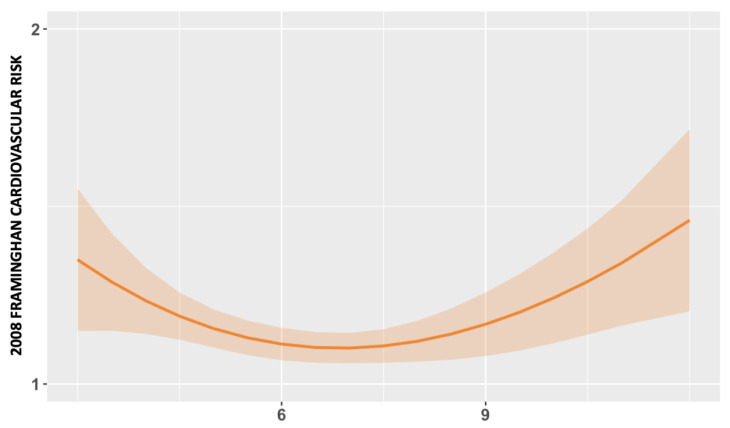
Third-degree polynomial multiple linear regression showing the association between sleep patterns (TST) and cardiovascular risk, according to the 2008 Framingham calculation.

Regarding CVD risk, according to Framingham’s calculator, the short sleep pattern group presented a median of 14.7% (IQR: 7.2–25.5) compared with the normal sleep pattern group’s 12.8% (IQR: 6.6–27.1), and those who belonged to the prolonged sleep pattern group presented 23.4% (IQR: 8.1–30.0) (Figure 3). The third-degree regression model revealed a U-shaped association between TST and the 2008 Framingham CVD risk (Figure 4).

## 4. Discussion

The main findings of this study are as follows: (A) self-reported sleep duration was associated with all-cause mortality (longitudinal analysis) and CVD risk (prevalent analysis); (B) both short and prolonged sleep durations were associated with negative outcomes; (C) after adjustment based on OSA, these associations remained significant.

This study has highlighted the importance of sleep duration regarding mortality risk. Several studies have confirmed the presence of a U-shaped association between sleep duration and mortality, indicating that individuals with both short and prolonged sleep durations have greater mortality risk than those with intermediate sleep durations. Poor sleep quality has been linked to a higher risk of CVD, including congestive heart failure, coronary heart disease, heart attacks, and strokes. Sleep-related disorders have also been associated with increased risks of CVD. Chronic inflammation is a potential mechanism of CVD, and controlling one’s intake of inflammatory food could reduce the 10-year CVD risk, especially for those with sleep disorders. Sleep duration is also considered a risk factor for cardiometabolic disease and the associated mortality. Therefore, sleep time is an important factor in mortality and CVD risk.

Even though those with normal sleep patterns exhibited cardiovascular risk, short and prolonged sleep patterns resulted in superior cardiovascular risk [28]. According to the AASM’s consensus, sleep hours for healthy adults (18–60 years) should be no less than 7 h per night, which has been inferiorly associated with a wide spectrum of cardiometabolic and mental diseases [29]. In particular, healthy sleep patterns appear to be associated with lower cardiovascular risk [30]. In the same context, the AHA published a scientific article [31] in which one of the objectives was to predict 10-year cardiovascular risk in a sample of 7.690 adults in the United States, aged between 40 and 79 years. This study reported an increase in cardiovascular risk in adults with short and prolonged sleep patterns compared with those with 7–8 h sleep patterns [31]. Recently, Zhong Q. et al. studied 45,919 participants, and their results suggested lower risk of cardiovascular diseases, such as coronary heart disease and stroke, in participants with better sleep patterns, especially those between 30 and 60 years old [30]. Similarly, a prospective study by Fan M. et al. that included 385.292 participants confirmed the findings of Zhong Q. et al., also adding that populations with low genetic susceptibility to cardiovascular risk could lose this characteristic in the absence of a healthy sleep pattern (early chronotype, 7–8 h sleep per day, no or rare insomnia, no snoring, and no frequent excessive daytime sleepiness) [32].

Our data show a significant association between long sleep patterns and cardiovascular risk (Model 2). Akinseye O.A. et al. published similar results, suggesting an association between abnormal sleep durations and an increased risk of stroke in diabetic patients [33]. A prolonged sleep pattern is characterized as ≥9–10 h [12]. Additionally, evidence has suggested that this pattern’s other effects include reductions in the ability to tolerate sleep pressure, more sleep fragmentation, less activity in the morning, minor average daily temperatures, and longer duration of melatonin secretion at night [13]. Moreover, prolonged sleep patterns are more associated with several cardiovascular consequences in the adult population [12] compared with sleep durations of 7–8 h [14]. As mentioned above, regarding sleep duration, the consensus has converged on a recommended mean of 7–8 h in the adult population [12]. However, exclusive consideration of sleep time and not sleep architecture would be inappropriate in a clinical context. For instance, those with short sleep patterns demonstrated more waking after sleep onset (WASO), longer REM stages, and similar or less slow-wave sleep compared with those who have long sleep patterns [13].

Our study has shown that both short and prolonged sleep pattern durations have significant associations with mortality risk. Several studies have suggested that the relationship between mortality and sleep duration has a significant interaction field [34,35,36]. Additionally, they have mentioned that short and prolonged sleep patterns play significant roles as predictors of death [37]. In fact, under objective measurements (8 h of PSG), the findings of Fernandez-Mendoza J, et al. (2020) suggested that short sleep patterns (<6 h) predict mortality prognoses in adults who have comorbidities such as hypertension, type 2 diabetes, heart disease, and stroke [38]. Similarly, sleeping for 9 or more hours has been associated with a 42% increase in mortality risk in women [39]. According to a review by Grandner M.A. et al., the factors of fatigue, sleep fragmentation, immune function, lack of challenge, underlying disease (sleep apnea, heart disease), photoperiodic abnormalities, and depression could explain the association between long sleep patterns and mortality [40]. However, Patel S.R. et al. suggested two general determinants: (i) psychiatric factors, such as depression, and (ii) socioeconomic factors, such as low socioeconomic status, lack of employment, low household income, and low perceived societal status. Short sleep patterns have been associated with unmarried people, lower socioeconomic status, and alcoholic behavior [41]. These associations with short and prolonged sleep patterns reinforce the idea of generation of multidisciplinary medical monitoring and early lifestyle education when these patterns are found in a clinical context. Indeed, the systematic review, meta-analysis, and meta-regression by Itani O. et al. suggested the importance of focusing future studies on psychoeducation or psychosocial intervention in the mentioned populations [41]. In our results, significant associations were observed with an adjusted model (based on baseline comorbidities, cardiovascular risk, and RDI) in patients with short sleep patterns and higher mortality risk. The existing scientific evidence has converged on the association between OSA and increased cardiovascular events such as heart attacks and coronary disease [15]. In addition, an association between OSA and increased cardiovascular risk and mortality has been suggested in the field [16,17]. OSA episodes are accompanied by hemodynamic imbalance and endothelial dysfunction due to sympathetic hyperactivity, increased blood pressure during sleep, hypoxic episodes that favor the presence of cardiac arrhythmia, changes in intrathoracic pressure, increased heart rates, sleep fragmentation, and arousal or microarousals that cause increased muscle tone in the oropharyngeal dilator muscles [42,43,44]. Regarding OSA and durations of sleep patterns, the findings of a study by Risso T.T. et al., in which the objective was to analyze sleep durations in OSA patients, showed that patients with short sleep patterns had higher AHI values (50.18 ± 30.86 events/h) compared with those who slept for about 7 h (23.21 ± 20.45 events/h) [45]. In addition, a study by Priou P. et al. suggested that a combination of severe OSA and a short sleep pattern will increase the risk of hypertension up to four times compared with that of normal sleepers who do not have OSA [46]. In this context, some studies have mentioned significant associations between visceral obesity metabolic syndrome and OSA patients with short sleep patterns [47,48].

The main limitation of this study is related to the characteristics of the sample, in which all of the subjects were Hispanic/Latino, although this study has increased the visibility of the crucial role of total hours of sleep in the adult population. In addition, this study was conducted at a sleep center, where most of the subjects had some type of sleep disorder. Finally, TSTs were recorded through sleep self-reporting, in the context of which the gold standard is wrist actigraphy accompanied by a sleep diary. Despite these limitations, this research has highlighted the key role of sleep duration and its associations with cardiovascular risk and mortality in the Hispanic/Latino population.

## 5. Conclusions

Among patients with short sleep patterns (≤6 h/night) and prolonged sleep patterns (≥9 h/night), there was an association with all-cause mortality and cardiovascular risk; after adjustment for respiratory event rates, this association remained significant for both sleep duration patterns (≤6 h/night and ≥9 h/night). Finally, a U-shaped association was found between the durations of short and long sleep patterns and increased cardiovascular risk at 10 years.

## Figures and Tables

**Table 1:** Baseline characteristics of the cohort (n = 1385). TSTs were categorized as short (≤ 6 h/night), normal (6 to 9 h/night), and long (≥9 h/night).

	Short TST (n = 632)	Normal TST (n = 639)	Long TST (n = 114)	*p*-Value
		Sociodemographic Data		
Sex				<0.001
Male, n (%)	515 (81.5%)	495 (77.5%)	72 (63.2%)	
Female, n (%)	117 (18.5%)	144 (22.5%)	42 (36.8%)	
Age (Years)	53.0 [43.0;63.0]	52.0 [41.0;63.0]	63.0 [43.0;73.8]	<0.001
BMI (Kg/m^2^)	29.5 [26.7;32.9]	29.5 [26.7;33.1]	30.2 [26.7;33.8]	0.607
		Comorbidities		
Smoking Status				0.659
Non-Smoker (%)	290 (46.0%)	311 (48.7%)	55 (48.2%)	
Current (%)	197 (31.3%)	191 (29.9%)	38 (33.3%)	
Former (%)	143 (22.7%)	136 (21.4%)	21 (18.4%)	
Systolic Pressure (mmHg)	120 [110;130]	120 [110;130]	124 [116;140]	0.087
Hypertension, n (%)	208 (37.5%)	181 (33.5%)	45 (51.1%)	0.005
Diabetes Mellitus, n (%)	172 (27.2%)	153 (24.0%)	34 (29.8%)	0.259
Framingham 2008 10-year CVD Risk, n (%)	14.7 [7.44;27.1]	11.8 [6.26;25.1]	23.4 [8.11;30.0]	0.023
Death, n (%)	59 (9.34%)	56 (8.76%)	31 (27.2%)	<0.001
		Sleep Apnea		
Severity of OSA				0.284
Non-OSA, n (%)	93 (14.7%)	90 (14.1%)	15 (13.2%)	
Mild OSA, n (%)	182 (28.8%)	169 (26.4%)	28 (24.6%)	
Moderate OSA, n (%)	167 (26.4%)	174 (27.2%)	23 (20.2%)	
Severe OSA, n (%)	190 (30.1%)	206 (32.2%)	48 (42.1%)	

Abbreviation list: BMI: body mass index; OSA: obstructive sleep apnea; CVD: cardiovascular disease. Statistically significant values are those where *p* < 0.05.

**Table 2:** Multivariable Cox regression analyses assessing the association of total sleep time (primary exposure) with all-cause mortality.

	Model 1 (HR, 95% CI)	Model 2 (HR, 95% CI)	Model 3 (HR, 95% CI)
Short TST	2.06 (1.36–3.2)	2.28 (1.36–3.84)	2.51 (1.48–4.25)
Normal TST (*)	---	---	---
Long TST	3.76 (1.92–7.36)	3.69 (1.43–9.51)	3.97 (1.53–10.29)

Cox regression models: Model 1: Unadjusted; Model 2: Adjusted for baseline cardiovascular risk, including age, gender, body mass index, diabetes mellitus, smoking, systolic blood pressure, and blood pressure medication; Model 3: Model 2 + rate of respiratory events. * Reference group.

## Data Availability

All data will be available through personal communication with the corresponding authors.
